# PAK4 suppresses TNF-induced release of endothelial microparticles in HUVECs cells

**DOI:** 10.18632/aging.103173

**Published:** 2020-07-12

**Authors:** Shouqin Zhang, Yingjie Yin, Congye Li, Yi Zhao, Qixing Wang, Xiangyu Zhang

**Affiliations:** 1 Department of Critical Care Medicine, Shanghai Tenth People’s Hospital, Tongji University School of Medicine, Jing’an, Shanghai, China; 2 Department of Critical Care Medicine, The Affiliated Hospital of Medical School of Ningbo, Jiangbei District, Ningbo, Zhejiang Province, China

**Keywords:** endothelial microparticle, PAK4, TNF, apoptosis, inflammation

## Abstract

Tumor necrosis factor-α (TNF) is a pro-inflammatory cytokine upregulated in many inflammatory diseases, and a potent inducer of endothelial cell-derived microparticle (EMP) formation. In this study, we identified the protein kinase PAK4 as a key regulator of the TNF-induced EMP release from human umbilical vein endothelial cells (HUVECs). TNF induces dose- and time-dependent EMP release and downregulation of PAK4 and upstream cdc42 in HUVECs. PAK4 suppression or inhibition of its kinase activity increases TNF-induced EMP release and apoptosis in HUVECs, while PAK4 overexpression reduces EMP release and apoptosis in TNF-stimulated cells. Collectively, these data indicate that PAK4 suppresses TNF-induced EMP generation occurring during apoptosis, and suggest that modulation of PAK4 activity may represent a novel approach to suppress the TNF-induced EMP levels in pro-inflammatory disorders and other pathological conditions.

## INTRODUCTION

Microparticles (MP) are small pro-inflammatory vesicles released by various cells. MP can be generated by nearly every cell type during activation, injury or apoptosis [1, 2]. They range in size from 0.1 to 1.1 μm, and contain a membrane, surface markers, and cytoplasmic and nuclear constituents of their original cells [3]. Once released into the circulation, MP bind and fuse with their target cells through receptor–ligand interactions, thereby acting as biological vectors mediating vascular inflammation and coagulation [4–6].

In response to apoptotic and other stimuli, vascular endothelial cells can release endothelial microparticles (EMP) [7]. Although EMP can be found in healthy individuals, their high plasma levels have been associated with numerous inflammatory diseases, including atherosclerosis, sepsis, multiple sclerosis and cerebral malaria, supporting their role as effectors and markers of vascular dysfunction [8–10]. Therefore, a better understanding of EMP formation and function may facilitate the development of therapies for vascular diseases.

Tumor necrosis factor-α (TNF) is a pro-inflammatory cytokine that has important roles in regulating cell proliferation and apoptosis, inflammation responses, and immune functions [11, 12]. Injection of TNF leads to a disordered endothelial barrier, increased pulmonary microvascular permeability, rearranged cytoskeleton, and increased circulating EMP levels [11, 13, 14]. Growing evidence shows that TNF is a model agent for *in vivo* and *in vitro* EMP formation.

In murine models of lung injury, increased levels of circulating EMP are accompanied by an inhibition of the cytoskeleton regulating protein PAK4 [13, 15], and have been proposed as an aspect of cellular dysfunction [16]. However, the relationships between EMP and PAK4 still remains unclear. p21-activated kinases (PAKs) are well known effector proteins of the Rho GTPase family [17]. PAK4 is a ubiquitously expressed essential group II PAK [18], a subfamily of serine/ threonine kinases [19]. PAK4 knock-out mice are embryonically lethal due to their defects in the fetal heart and in neuronal development [20]. There is also an evidence showing that PAK4 promotes tumorigenesis and is oncogenic when overexpressed [21–23]. As the effector of the Rho GTPase cdc42, PAK4 controls the cytoskeleton primarily through the regulation of polymerized actin structures, particularly the formation of filopodia and lamellipodia, but can also act upon microtubule organization [17].

In this study, we analyzed the relationship between PAK4 and EMP formation in human umbilical vein endothelial cells (HUVECs). Our results indicate that PAK4 suppresses TNF-induced EMP generation that occurs during apoptosis in HUVECs.

## RESULTS

### TNF induces EMP release both in HUVECs and in mice

**Figure 1:**
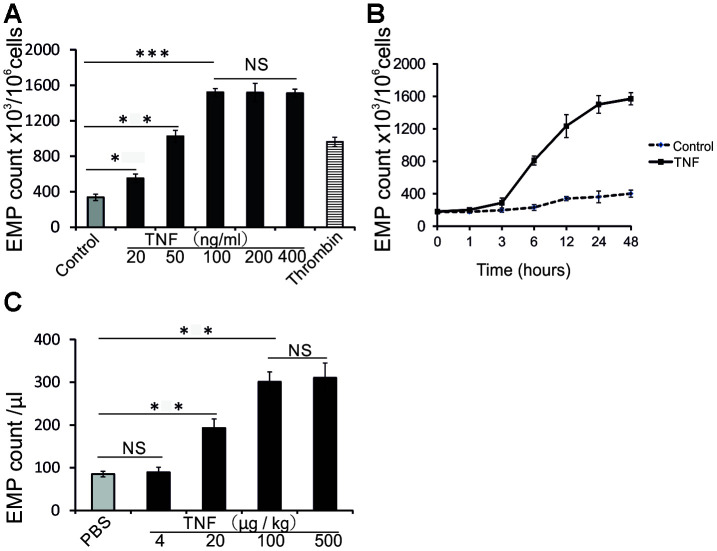
**TNF induces EMP release *in vitro* and *in vivo*.** (**A**) EMP release analyzed by flow cytometry (FC) in HUVECs treated 24 h with increasing TNF concentrations, or thrombin (2 IU/ml). The data represent the mean values based on 1x10^6^ cells; three independent experiments were performed. (**B**) EMP release analyzed by FC in untreated HUVECs, or HUVECs treated with 100 ng/ml TNF for indicated times. The data represent the mean values of EMP per 1x10^6^ cells from three independent experiments. (**C**) EMP release analyzed by FC in sera of TNF-treated mice. Male C57BL/6 mice (10 weeks old) were randomly assigned to 5 groups (n=10 mice per group) and treated with PBS or increasing doses of TNF for 24 hours. The data represent the mean values of EMP/μl; * P<0.05, ** P<0.01, *** P<0.001, NS indicates no significance.

First, we analyzed the ability of TNF to induce the EMP release *in vitro* and *in vivo*. *In vitro*, the ability of TNF to induce EMP was analyzed in human umbilical vein endothelial cells (HUVECs) by flow cytometry using annexin V-FITC, which binds to phosphatidylserine exposed on EMP. As shown in Figure 1A, TNF between 20 ng/mL and 100 ng/mL, dose-dependently induced the EMP release in comparison with untreated control cells; stimulation with a serine protease, thrombin, was used as a positive control. The level of released EMP in response to thrombin was about half of that observed in response to 100 ng/ml TNF (Figure 1A). The release of EMP was also time dependent, with a significant increase between 3 and 24 hours (Figure 1B). Thus, the following experiments with HUVECs were performed using a 24 h stimulation with 100 ng/mL TNF.

Next, we investigated whether TNF induces EMP release also in vivo. Male C57BL/6 mice (10 weeks old) were randomly assigned to 5 groups (n=10) and treated 24 h with PBS or increasing doses of TNF (4, 20, 100, or 500 μg/kg). As shown in Figure 1C, EMP in mice sera increased significantly when treated with 20 and 100 μg/kg TNF.

### TNF induces cytoskeleton rearrangement and down-regulates PAK4

**Figure 2:**
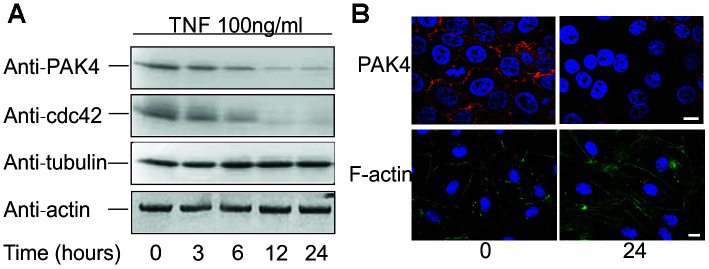
**TNF down-regulates PAK4 and cdc42 expression in HUVECs.** (**A**) Western blotting of HUVECs incubated with 100 ng/ml TNF for up to 24 hours, analyzed using anti-PAK4, anti-cdc42, anti-β-actin, and control anti-tubulin antibodies. (**B**) Confocal microscopy of HUVECs incubated 0 and 24 h with 100 ng/ml TNF, and stained for PAK4 and F-actin; scale bar = 10 μm. Cells were counter stained with DAPI.

EMP shed by endothelial cells are associated with cytoskeleton disorders [15]. Thus, PAK4 as a regulator of cytoskeletal dynamics and cell motility [19] may also play a role in EMP generation. To test this hypothesis, we treated HUVECs with 100 ng/ml TNF for 24 hours, and stained the cells with Phalloidin-FITC or anti-PAK4 polyclonal antibody. Without TNF treatment, the F-actin bundles mainly localized at cell border, but in TNF-treated cells, the F-actin bundles were throughout the cells, and the dense peripheral actin band was replaced by thicker stress fibers (Figure 2B, lower panel). In untreated cells, PAK4 could be observed at the cell-cell contacts, while after TNF stimulation, PAK4 staining could hardly be detected (Figure 2B, upper panel). Immunoblotting indicated a time dependent decrease in the approximately 64 kDa PAK4 protein band, and the upstream cdc42 protein (Figure 2A). Although F-actin rearrangement was observed by confocal microscopy, TNF did not change the total levels of β-actin (Figure 2A); tubulin was used as a loading control. These data demonstrate that TNF down-regulates PAK4 in HUVECs.

**Figure 3:**
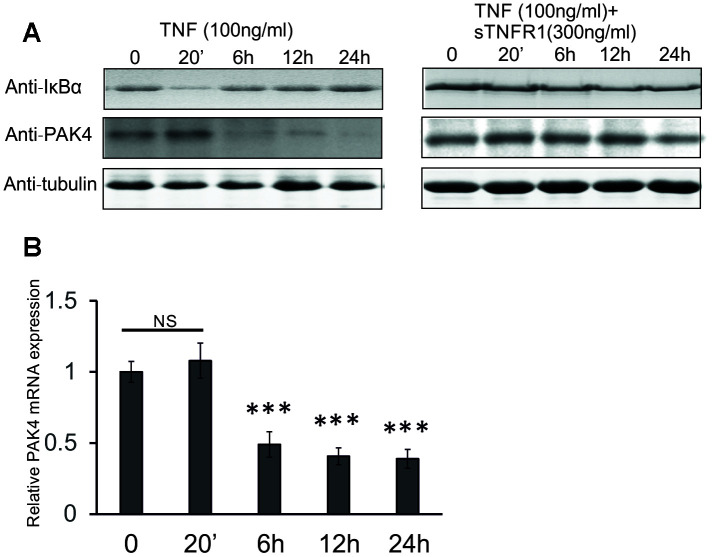
**TNF decreases PAK4 gene and protein levels in HUVECs.** (**A**) Western blot analysis of PAK4 and IκBα in HUVECs stimulated with 100 ng/ml TNF without and with 300 ng/ml soluble TNFR1. (**B**) Quantitative real-time PCR of PAK4 mRNA in HUVECs treated with 100 ng/ml TNF. All data are expressed as the mean + SD; *** P<0.001, compared with untreated cells.

To investigate how TNF down-regulates the PAK4 levels, HUVECs were treated with TNF alone, or with TNF + soluble TNFR1, the soluble part of TNF receptor 1 that interacts with TNF and inhibits its binding to TNFR1 on the cell membrane. After 6 h stimulation with TNF, the PAK4 expression decreased, both at protein and mRNA levels (Figure 3A left panel, and Figure 3B). However, soluble TNFR1 stabilized the PAK4 protein levels in TNF-treated cells (Figure 3A, right panel). In addition, TNF induced a rapid degradation of IκBα, the inhibitor of NF-κB, in HUVECs (Figure 3A, right panel), but this degradation was prevented by the soluble TNFR1 (Figure 3A, left panel). These results indicate that TNF decreases PAK4 expression through the TNFR1-dependent pathway that might also involve NF-κB signaling.

### Involvement of PAK4 in TNF-induced EMP release

**Figure 4:**
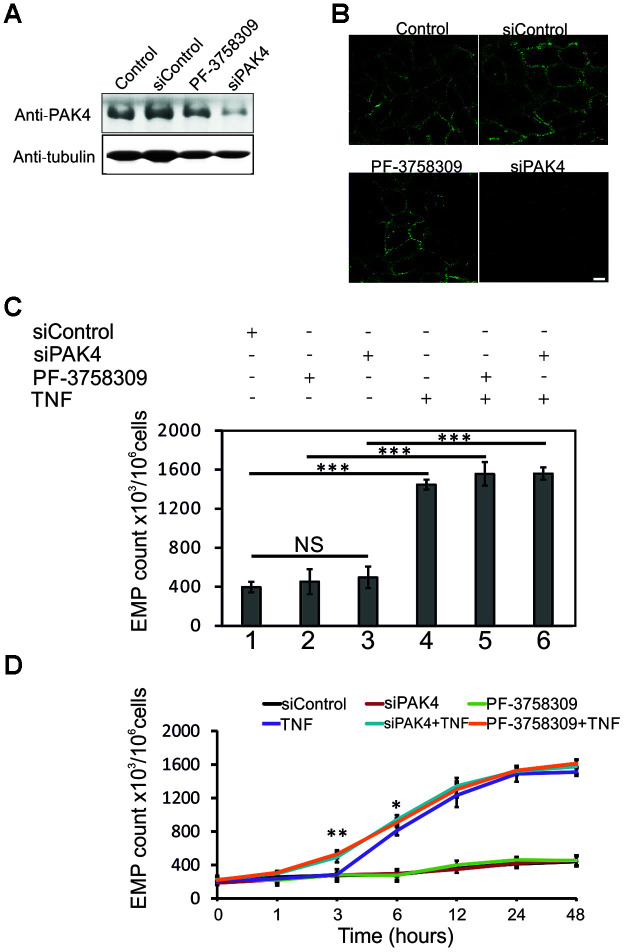
**PAK4 inhibition sensitizes HUVECs to TNF-induced EMP release.** (**A**) Western blotting of HUVECs transfected 48 h with PAK4 or control siRNAs, or treated 24 h with PAK4 inhibitor PF-3758309, and analyzed by PAK4 and control tubulin antibodies. (**B**) Immunofluorescence of HUVECs transfected with PAK4 or control siRNAs, or treated with PF-3758309, and stained by anti-PAK4 antibody; scale bar = 10 μm. (**C**) EMP release measured by FC in HUVECs transfected 48 h with PAK4 or control siRNAs, or treated 24 h with PF-3758309, and stimulated 24 h with TNF (100 ng/ml). (**D**) Time course of EMP release in TNF-treated HUVECs transfected 48 h with PAK4 or control siRNAs, or incubated 24 h with PF-3758309; * P<0.05, ** P<0.01.

To investigate the role of PAK4 in TNF-induced EMP generation, we first inhibited PAK4 function in TNF-stimulated (100 ng/ml) HUVECs by PAK4 inhibitor PF-3758309 or PAK4 siRNAs. PF-3758309 is an ATP-competitive, small-molecule pyrrolopyrazole inhibitor, blocking the PAK4 kinase activity and downstream pathways [24]. HUVECs were treated with 10 μM PF-3758309 overnight or transfected with PAK4 siRNAs for 48 h, before TNF stimulation. To stably knockdown PAK4 and avoid off-target effects, two distinct small interfering RNAs targeting PAK4 were used. As shown in Figure 4A, PF-3758309 had no obvious effect on PAK4 expression, but PAK4 specific siRNAs effectively reduced the PAK4 protein levels in HUVECs, with an estimated efficiency of ~85% knockdown (immunoblotting quantification using ImageJ). Immunofluorescent staining of PAK4 in PF-3758309 or PAK4 siRNAs treated cells showed similar results (Figure 4B).

Without TNF stimulation, PF-3758309 or PAK4 siRNAs treatment did not show any significant effect on the basal EMP release, compared to siRNA control (Figure 4C, lanes 1, 2, 3). TNF (24 h) stimulation significantly increased the EMP release, but this release was not substantially affected by PF-3758309 or PAK4 suppression by siRNA (Figure 4C, lanes 4, 5, 6). Next, we examined the time dependent EMP release upon 100 ng/ml TNF stimulation in PF-3758309 or PAK4 siRNAs treated HUVECs. Three hours after TNF treatment, PAK4 knockdown (siPAK4+TNF) or PAK4 inhibition (PF-3758309+TNF) markedly increased the EMP release (Figure 4D, blue and orange curves vs purple curve, P<0.01), indicating that PAK4 knockdown or inhibition sensitizes HUVECs to TNF-induced EMP release.

### Overexpression of PAK4 decreases TNF-induced EMP release

**Figure 5:**
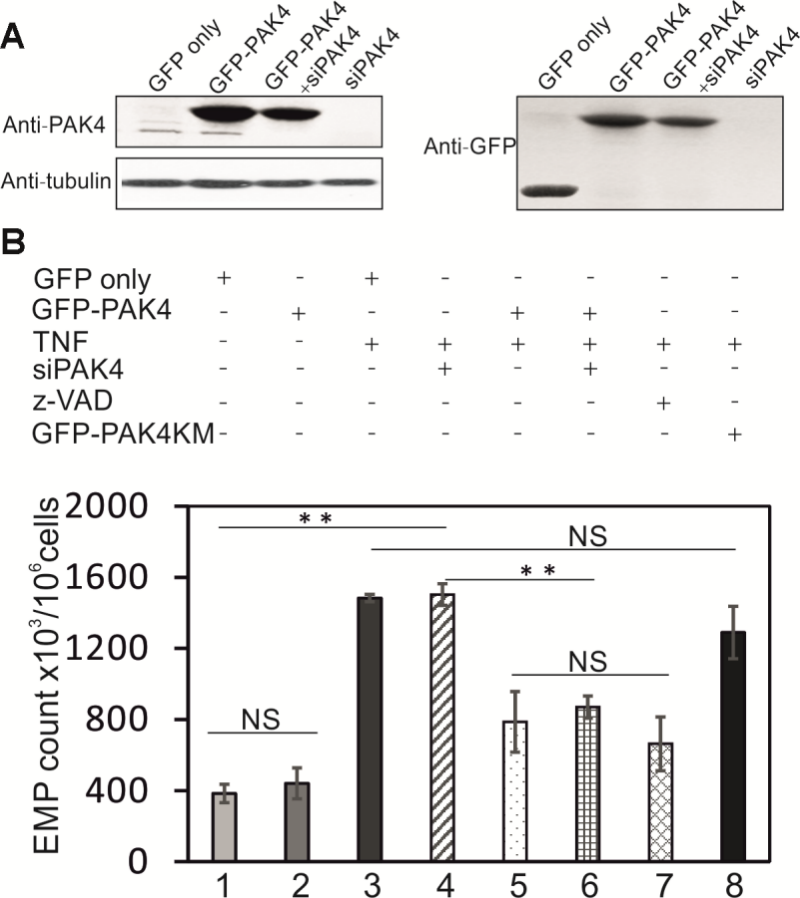
**PAK4 overexpression decreases TNF-induced EMPs release.** (**A**) Western blotting of HUVECs transfected 48 h with GFP, GFP-PAK4 plasmid, siPAK4 or GFP-PAK4+siPAK4, and analyzed by PAK4 and control tubulin antibodies. (**B**) EMP release in HUVECs transfected with GFP, GFP-PAK4, GFP-PAK4KD, siPAK4 or GFP-PAK4+siPAK4, and stimulated 24 h with TNF (100 ng/ml) or control vehicle. Overexpression of GFP-PAK4 significantly inhibits TNF-induced EMP release (lane 5 vs lane 3). The TNF-induced EMP is also inhibited by caspase inhibitor z-VAD (lane 7). PAK4 kinase mutant (GFP-PAK4KM) does not reduce the TNF-induced EMP release (lane 8). The values represent the mean of three independent experiments. ** P<0.01; NS, not significant.

To determine whether activation of PAK4 decreases the TNF-induced EMP release, we transiently overexpressed GFP-tagged PAK4 in HUVECs. Western blot analysis revealed a highly expressed GFP-PAK4 protein band in addition to the endogenous PAK4 band (Figure 5A). Overexpression of GFP-PAK4 reduced the TNF-induced EMP release (Figure 5B, lane 5 vs lane 3, P<0.01), but had no effect on the basal EMP release (Figure 5B, lane 2 vs lane 1). However, in contrast to PAK4, the PAK4 kinase mutant (GFP-PAK4KM) did not significantly decrease the TNF-induced EMP release (Figure 5B, lane 8 vs lane 3). Pre-incubation of HUVECs with z-VAD-FMK (2 μM), a pan-caspase inhibitor, was sufficient to abrogate the EMP release induced by TNF (Figure 5B, lane 7 vs lane 3, P<0.01). The effect of z-VAD-FMK on TNF-induced EMP release was similar to the overexpressed PAK4 (Figure 5B, lane 7 vs lane 5). Overexpression of GFP-PAK4 also markedly prevented the EMP release induced by TNF in PAK4 knockdown cells (Figure 5C, lane 6 vs lane 4, P<0.01).

### PAK4 prevents TNF-induced caspase activation and apoptosis

**Figure 6:**
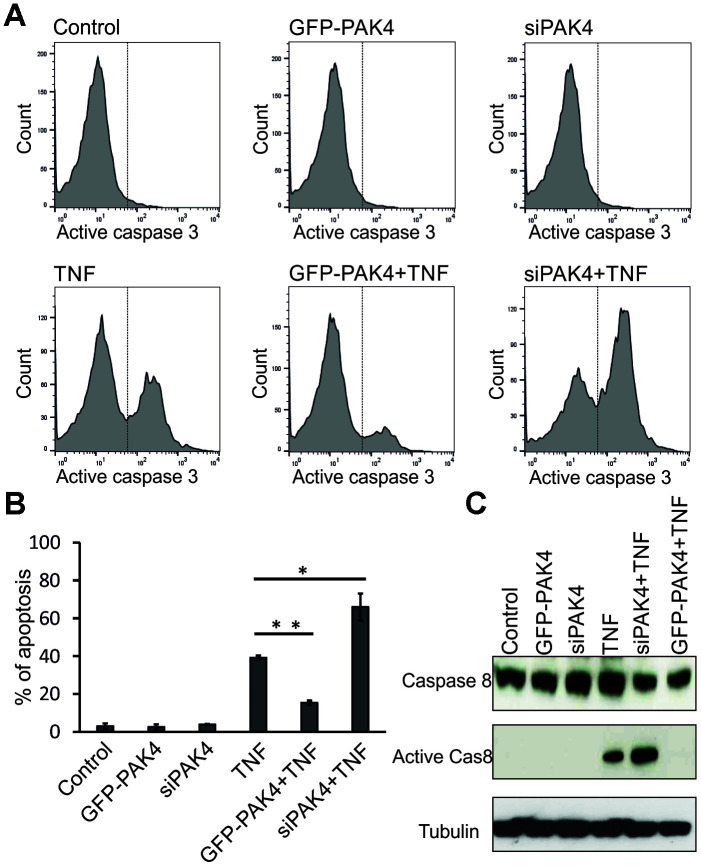
**PAK4 inhibits caspase activation and apoptosis in HUVECs.** (**A**) Active caspase-3-FITC staining analyzed by FC in HUVECs transfected 48 h with GFP-PAK4 or PAK4 siRNA, and stimulated with TNF (100 ng/ml). (**B**) Quantitative evaluation of apoptotic cells analyzed by FC in panel A. The data represent the mean ± s.d. of three independent experiments. P-values were calculated using Student’s t-test. * P<0.05, ** P<0.01. (**C**) Western analysis of total caspase-8, active caspase-8, and control tubulin in TNF-stimulated HUVECs transfected with GFP-PAK4 or PAK4 siRNA.

Caspases mediate the membrane blebbing associated with apoptosis [25]. Since the TNF-induced EMP release was reduced by the caspase inhibitor z-VAD-FMK (Figure 5C, lane 5), we examined the PAK4 involvement in caspase activation and apoptosis. GFP-PAK4 or PAK4 siRNA were transiently transfected into HUVECs and after 48 h, cells were treated with 100 ng/ml TNF overnight. Flow cytometry analysis using FITC-conjugated active caspase-3 antibody revealed that GFP-PAK4 or PAK4 siRNA transfection had no effect on active caspase-3 in the absence of TNF (Figure 6A, upper panels). In the presence of TNF, about 40% of un-transfected cells stained as active caspase-3 positive; however, GFP-PAK4 overexpression reduced the percentage of active caspase-3 positive cells to about 15%, and PAK4 knockdown increased it to about 66%, (Figure 6A, lower panels). The TNF-induced apoptosis was significantly reduced by PAK4 overexpression, but increased by PAK4 suppression (Figure 6B). In addition, PAK4 suppression in TNF-treated cells increased the activation of caspase-8 analyzed by immunoblotting (Figure 6C).

## DISCUSSION

In this study, we found that TNF induces EMP release in a dose and time dependent way in HUVECs. Under TNF stimulation, cdc42 and its effector, PAK4 protein levels were down regulated. PAK4 suppression increased the EMP release and apoptosis in TNF-stimulated HUVECs, while PAK4 overexpression reduced the EMP release and apoptosis in TNF-stimulated cells. These data suggest that PAK4 suppresses the EMP generation and apoptosis induced by TNF in HUVECs.

EMP are plasma membrane particles that are shed from activated or apoptotic endothelial cells and retain many of the ligands and receptor proteins expressed by the original cells [7, 11]. Even though EMP are present in healthy individuals, they are elevated in patients with inflammatory diseases [5, 26] and sepsis [27]. EMP release can be stimulated by pro-inflammatory cytokines, oxidative stress, or infectious agents [7, 11]. Recent studies suggest a link between endothelial damage, EMP release, and the modulation of inflammatory and immune responses.

TNF, together with other cytokines, induces endothelial expression of adhesion molecules and augments monocyte infiltration [28]. TNF also initiates apoptosis in endothelial cells [29]. TNF blood levels in patients with inflammatory diseases positively correlate with the levels of circulating MP derived from various cells, including endothelial cells, erythrocytes, monocytes and platelets [30, 31]. *In vivo* and *in vitro* studies have shown that TNF is a model agent for EMP formation [7, 11, 32]. However, the exact mechanism by which TNF induces the EMP release has not been characterized. In this study, we show that TNF induces EMP in HUVECs in a dose and time dependent manner (Figure1).

The p21-activated kinase 4 (PAK4) belongs to the serine/threonine kinase family, and serves as a target for the Rho GTPase cdc42 [19]. PAK4 has important roles in regulating cell adhesion, cytoskeleton remodeling, embryonic development, and oncogenesis [15, 20–22]. In addition, PAK4 was reported to protect cells from apoptosis by phosphorylating the pro-apoptotic protein Bad [33]. Micropartical formation is a common mechanism of membrane shedding by activated or apoptotic cells, requiring cytoskeleton rearrangement [1, 4, 26]. TNF induced EMP through the cdc42/PAK4 pathway is consistent with a role of Rho-GTPases in the cytoskeletal changes leading to endothelial blebbing [6, 34]. Our findings demonstrate the involvement of PAK4 in this pathway: TNF significantly reduces the PAK4 protein levels in HUVECs, and inhibition of PAK4 kinase activity or PAK4 suppression sensitize HUVECs to TNF-induced EMP release, while PAK4 overexpression reduces the TNF-induced EMP release.

Attenuated NF-κB survival signals may lead to a decrease in PAK4 expression. Our results indicate that NF-kB signaling might be involved in the TNF-induced downregulation of PAK4 in HUVECs. This is supported by previous studies that have suggested that PAK4 may act as a switch between NF-κB survival signaling and caspase-8-mediated apoptosis induced by TNF in hepatocarcinoma cells [35]. Decreased nuclear levels of NF-κB p65 were found in PAK4 knockdown cells [35, 36]. In addition, a previous study suggested that constitutive activation of NF-κB might compensate for the lack of PAK4 [12].

In conclusion, our data provide evidence that TNF induces a dose and time dependent release of EMP in HUVECs, by cdc42/PAK4 dependent pathway. Using gene expression and gene silencing, we identified PAK4 as a critical regulator of EMP generation by TNF. TNF downregulates the cdc42/PAK4 levels. Our data indicate that PAK4 suppresses the TNF-induced EMP generation and apoptosis in HUVECs through the TNFR1 signaling pathway. Since the increased EMP levels have been reported in multiple pro-inflammatory and pathologic conditions including sepsis [27], cardiovascular disease [37], thrombosis [5], angiogenesis, inflammatory response, and hypercoagulability [26], our data suggest that modulation of PAK4 activity might represent a novel approach to suppress the TNF-induced EMP levels in these pathological conditions.

## MATERIALS AND METHODS

### Animals

Male C57BL/6 mice (weight about 25-29g, 10 weeks old) were kept under a conventional 12 hour light-dark cycle in a temperature-controlled room with free access to water and food. Mice were randomly assigned to 5 groups (10 mice per group) and treated with PBS or different doses of TNF (4 μg/kg, 20 μg/kg, 100 μg/kg or 500μg/kg) for 24 hours. All experimental protocols were approved by the Institutional Animal Care and Use Committee of the Shanghai Tongji University and performed in accordance with the National Institutes of Health Guidelines for the use of experimental animals.

### Cell culture

The primary human umbilical vein endothelial cells (HUVECs) were purchased from ATCC (PCS-100-010) and were grown in vascular cell basal media supplemented with endothelial cell growth kit components.

### Antibodies, proteins and drugs

Primary antibody against PAK4 was purchased from Proteintech. IκBα antibody was from Cell Signalling. Antibodies against tubulin, cdc42, β-actin, and IκBα, phalloidin-FITC, and horseradish peroxidase (HRP)-conjugated secondary antibody were purchased from Sigma. Alexa 488 and 568-conjugated secondary antibodies were from Molecular Probes. Caspase-8 and active caspase-8 antibodies were from Cell Signalling. Purified TNF protein and sTNFR1 (soluble part of TNFR1) were purchased from Immunotools (Friesoythe, Germany); thrombin was from Sigma. The PAK4 inhibitor PF-3758309 (Pfizer) was used at 10 μM.

### Measurements of EMP

Untreated or TNF treated HUVECs (7x10^5^ cells in 12-well plates) were collected by centrifugation (5000g for 5 minutes). EMP was measured in supernatants by flow cytometry (Beckman Coulter, Fullerton, CA). 10 μl of centrifuged media or sera were labelled by annexin V-FITC and 1 μm latex beads (Beckman Coulter, flowcount) were used as gating parameters. Particles less than or equal to 1 μm were defined as EMPs. Number of EMP was calculated as described5.

### HUVEC transfections

For transfection, HUVEC cells were seeded at 85%~90% confluency and allowed to adhere overnight. Small interfering RNA (siRNA), GFP-PAK4, or GFP-PAK4KM plasmid were transfected using Lipofectamine 2000 (Invitrogen) as described by the manufacturers. For studies of RNA interference, transfections were performed with *PAK4* short interfering (si) RNA duplexes (two different siRNAs mixed, final concentration 50 nM, Life Technologies) or a nonsilencing siRNAs as negative controls (Life Technologies). At 48 hours after siRNA transfection, cells were harvested to determine the level of PAK4 proteins by immunoblotting, or were stimulated with 100 ng/mL TNF.

### Cloning strategy

Human PAK4 DNA fragment was amplified from cDNA bank by PCR and cloned into pGEMTeasy (Promega) vector. GFP-PAK4 was cloned into the EcoRI/SalI site of EGFP-C2 vector. The GFP fusion protein was generated to carry GFP at its N-terminus. GFP-PAK4KM was cloned into the EcoRI/SalI site of EGFP-C2 vector. This PAK4 kinase mutant contains a lysine to methionine mutation at amino acid 350. The mutation was generated using site-directed mutagenesis kit (Stratagene).

### Immuno-fluorescence microscopy

HUVECs were cultured to subconfluence on 8 chamber glass slides (BD Falcon, Bedford, MA). After treatment, they were fixed in 4% paraformaldehyde (PFA) in phosphate-buffered saline (PBS) and permeabilized with 0.1% Triton X-100. Actin cytoskeleton was stained with Phalloidin-FITC. Cells were incubated with 1:400 diluted polyclonal anti-PAK4 antibody, followed by a sequential incubation with a 1:1000 diluted Alexa 488 or 568-conjugated secondary antibody. Nuclei were counter stained with DAPI (Sigma-Aldrich). The cells were imaged using a ZEISS 780 inverted confocal laser scanning microscope (ZEISS, Cambridge, UK) and analyzed by using Zen software.

### Western blotting

Cells were washed with cold PBS and lysed with 1 ml RIPA buffer (NEB, Hertfordshire, UK) containing protease inhibitor cocktail (Roche) on ice. Homogenized samples were microcentrifuged at full speed for 15 min at 4°C. The supernatants were subjected to SDS-PAGE. Separated proteins were transferred onto nitrocellulose membrane and probed with primary antibodies and HRP-conjugated secondary antibodies.

### Quantitative real time PCR

Total RNA was extracted from cultured HUVECs using Trizol solution (Invitrogen) and reverse transcribed into cDNA with a Reverse Transcriptase kit (Invitrogen) according to manufacturer’s instructions. The expression of PAK4 were detected using a TagMan mRNA assay kit (Biosystems). The relative expression was determined by the 2^-ΔΔCt^ method with GAPDH as the internal reference. Samples were analysed in triplicate. The primer sequences used were the following. PAK4-F: ATGTGGTGGAGATGTACAACA, PAK4-R: GTTCATCCTGGTGTGGGTGAC, GAPDH-F: GGAGCGAGATCCCTCCAAAAT, GAPDH-R: GGCTGTTGTCATACTTCTCATGG

### Apoptotic assay

Cells were fixed with the Cytofix/Cytoperm fixation and permeabilization solution kit (BD Pharmingen), stained with FITC-conjugated anti-caspase-3 antibody (BD Pharmingen), and analyzed using CyAn flow cytometer (Beckman Coulter, Fullerton, CA). Data were evaluated using Flowjo (Tree Star Inc., Ashland, Oregon).

### Statistics

Results are expressed as mean ± SD. The statistical significance was assessed using analysis of variance (ANOVA), followed by Student’s t-test. A P value of <0.05 was considered significant.
